# A decrease in NAD^+^ contributes to the loss of osteoprogenitors and bone mass with aging

**DOI:** 10.1038/s41514-021-00058-7

**Published:** 2021-04-01

**Authors:** Ha-Neui Kim, Filipa Ponte, Aaron Warren, Rebecca Ring, Srividhya Iyer, Li Han, Maria Almeida

**Affiliations:** 1 Division of Endocrinology, University of Arkansas for Medical Sciences, Little Rock, AR USA; 2 Department of Orthopaedic Surgery, University of Arkansas for Medical Sciences, Little Rock, AR USA; 3 Present Address: Department of Orthopedics, University of Colorado School of Medicine, Aurora, CO USA

**Keywords:** Osteoporosis, Senescence

## Abstract

Age-related osteoporosis is caused by a deficit in osteoblasts, the cells that secrete bone matrix. The number of osteoblast progenitors also declines with age associated with increased markers of cell senescence. The forkhead box O (FoxO) transcription factors attenuate Wnt/β-catenin signaling and the proliferation of osteoprogenitors, thereby decreasing bone formation. The NAD^+^-dependent Sirtuin1 (Sirt1) deacetylates FoxOs and β-catenin in osteoblast progenitors and, thereby, increases bone mass. However, it remains unknown whether the Sirt1/FoxO/β-catenin pathway is dysregulated with age in osteoblast progenitors. We found decreased levels of NAD^+^ in osteoblast progenitor cultures from old mice, associated with increased acetylation of FoxO1 and markers of cell senescence. The NAD^+^ precursor nicotinamide riboside (NR) abrogated FoxO1 and β-catenin acetylation and several marker of cellular senescence, and increased the osteoblastogenic capacity of cells from old mice. Consistent with these effects, NR administration to C57BL/6 mice counteracted the loss of bone mass with aging. Attenuation of NAD^+^ levels in osteoprogenitor cultures from young mice inhibited osteoblastogenesis in a FoxO-dependent manner. In addition, mice with decreased NAD^+^ in cells of the osteoblast lineage lost bone mass at a young age. Together, these findings suggest that the decrease in bone formation with old age is due, at least in part, to a decrease in NAD^+^ and dysregulated Sirt1/FoxO/β-catenin pathway in osteoblast progenitors. NAD^+^ repletion, therefore, represents a rational therapeutic approach to skeletal involution.

## Introduction

Loss of bone mass is a major cause for the occurrence of osteoporotic fractures in the aged population^1–3^. It is well documented that trabecular bone mass and cortical thickness decrease and intracortical porosity increases with age, with particular incidence in women^4–6^. The integrity of bone is maintained by a process known as bone remodeling in which teams of osteoclasts—giant multinucleated cells of the hematopoietic lineage—resorb the bone matrix which is then replaced by teams of osteoblasts—cells of mesenchymal lineage that secrete bone matrix^7^. With aging the number of osteoblasts slowly declines leading to unbalanced remodeling and loss of bone mass^8^. Much similar to humans, mice lose trabecular and cortical bone mass with age due to unbalanced remodeling and develop cortical porosity^9–12^. The decrease in bone formation with age is associated with lower osteoprogenitor numbers^13^; nonetheless, the cellular and molecular mechanisms responsible for skeletal aging remain unclear.

In several tissues the oxidized form of nicotinamide adenine dinucleotide (or NAD^+^), a co-enzyme that functions as an electron acceptor, decreases with aging^14,15^. Importantly, NAD^+^ precursors such as nicotinamide mononucleotide (NMN) or nicotinamide riboside (NR) exert anti-aging effect in tissues like the eye, muscle, vasculature, and pancreas^16,17^. NAD^+^ also increases longevity in different species such as worms, rats, and mice. Thus, a decline in NAD^+^ is a bona fide hallmark of aging. Most of the cellular NAD^+^ is recycled from nicotinamide NAM via the NAD salvage pathway. First, nicotinamide phosphoribosyl-transferase (*NAMPT*) converts NAM into NMN, which in turn leads to the production of NAD^+^ in a reaction catalyzed by nicotinamide mononucleotide adenylyl-transferase (*NMNAT*). Raising NAD^+^ levels prevents several age-associated events such as DNA damage, mitochondria dysfunction, cell senescence, and stem cell loss in different tissues in mice^17^. Some of these effects are mediated via the NAD-dependent Sirtuin1 (Sirt1).

Genetic evidence from our group and others has shown that Sirt1 promotes bone formation and the accrual of normal bone mass via action in osteoprogenitors^18,19^. Furthermore, long-term administration of Sirt1 activators to mice attenuates skeletal aging^20,21^. In osteoblast progenitors, Sirt1 deacetylates FoxOs and β-catenin and, thereby, stimulates Wnt signaling and osteoblastogenesis^18,19^. We have shown that an increase in mitochondrial reactive oxygen species (ROS) in cells of the mesenchymal lineage, including stem cells, osteoblast progenitors osteoblasts, and osteocytes, contribute to the decrease in bone formation with aging^22^. ROS stimulate FoxOs and promote the binding of FoxOs to β-catenin and, thereby, decrease osteoblastogenesis^23–25^. Importantly, FoxOs in osteoprogenitors are major inhibitors of bone formation^26^. These indirect lines of evidence support the idea that the Sirt1/FoxO/β-catenin pathway in osteoblast progenitors contributes to skeletal aging. However, whether this pathway is altered with aging remains unclear.

Here, we found that FoxO and β-catenin acetylation increase with age in osteoblast progenitors due to a decline in NAD^+^ levels. Using pharmacological and genetic tools to manipulate NAD^+^ levels, we provide data to suggest that a decline in NAD^+^ in osteoprogenitor cells contributes to skeletal aging.

## Results

### FoxO acetylation increases and NAD^+^ levels decrease with aging in osteoblastic cells

**Fig. 1:**
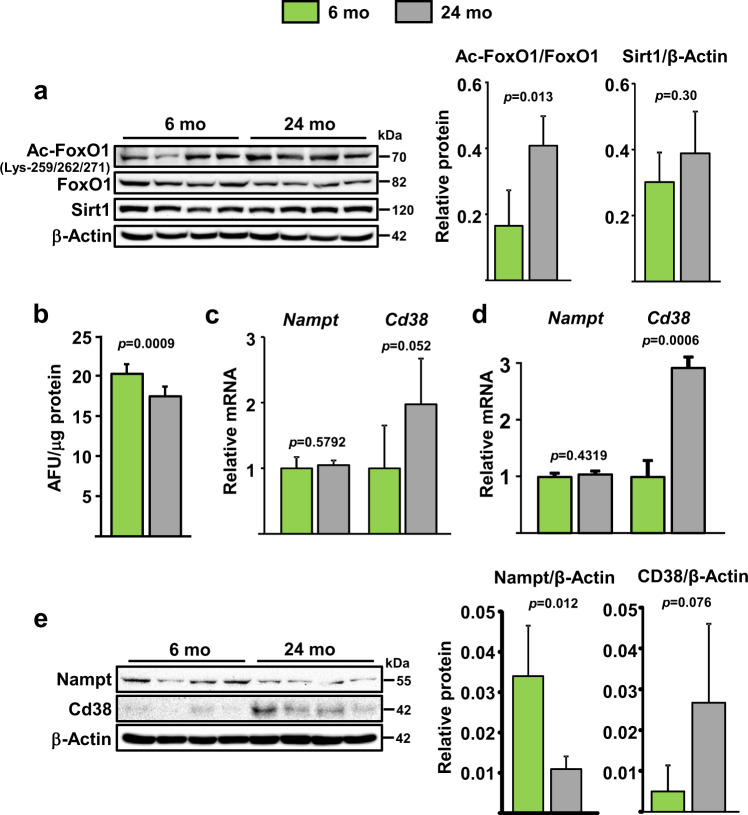
NAD+ declines with age in bone marrow osteoblastic cells. **a** Protein levels by western blot (left) and relative quantification (right) of band intensity in bone marrow stromal cells from 6- and 24-month-old C57BL/6 female mice cultured with ascorbic acid and β-glycerophosphate for 5 days; each lane represents one mouse (*n* = 4/group). **b** Relative cellular NAD^+^ levels in cultured osteoblastic cells pooled from 5 mice/group (triplicates of pooled cultures). AFU arbitrary fluorescence units. **c**, **d** mRNA levels by qRT-PCR in (**c**) CD45^−^ cells freshly isolated from 6- and 24-month-old C57BL/6 female mice (*n* = 5/group) and **d** cultured cells described in **a**. **e** Protein levels in cultured stromal cells described in **a**. Bar graphs represent mean ± SD, data analyzed by Student’s *t*-test.

We first examined the status of FoxO acetylation and levels of Sirt1 in bone marrow-derived osteoprogenitor cell cultures from 6- and 24-month-old C57BL/6 mice. Acetylation of FoxO1 was higher in cells from old mice while Sirt1 was unaffected (Fig. 1a). Because NAD^+^ levels influence Sirt1 activity, we quantified NAD^+^ levels and found that these were decreased in cells from old mice (Fig. 1b). Altered expression of NAD-synthesizing enzymes such as Nampt or consuming enzymes such as Sirtuins and Cd38 can cause changes in NAD^+^^14^. Freshly isolated bone marrow CD45^−^ cells or cultured bone marrow stromal cells from old mice had higher *Cd38* mRNA (Fig. 1c, d). The protein levels of Cd38 were also elevated in the cultured cells from old mice (Fig. 1e). *Nampt* mRNA was unchanged in bone marrow cells (Fig. 1c, d). However, the protein levels of Nampt were decreased with age (Fig. 1e). These findings suggest that both an increase in Cd38 and a decrease in Nampt contributes to the decline in NAD^+^ levels in osteoprogenitor cells.

### Nicotinamide riboside improves osteoblastogenesis in cells from old mice

**Fig. 2:**
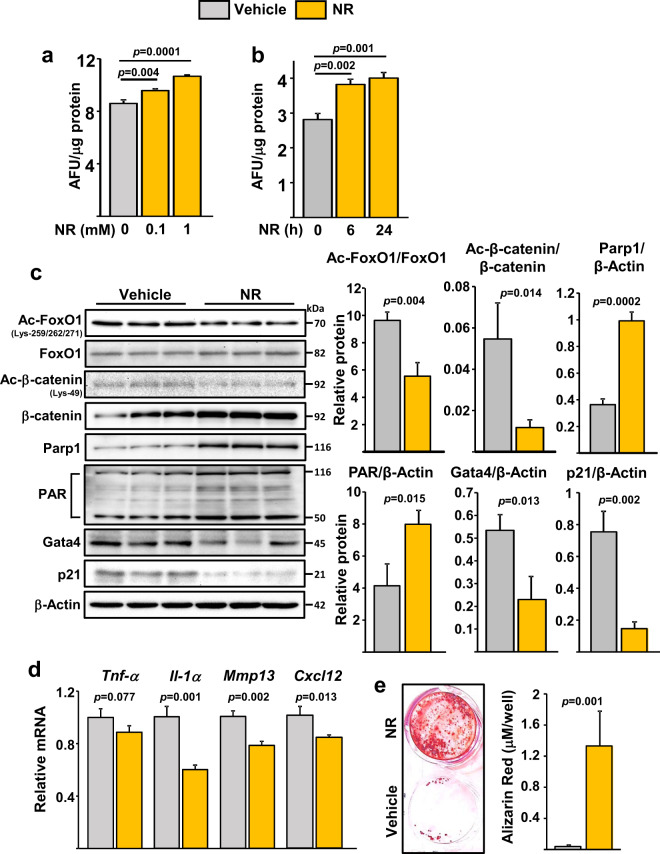
NR attenuates cell senescence and defective osteoblastogenesis in cultured bone marrow cells from old mice. **a**, **b** NAD^+^ levels in bone marrow stromal cells from 24-month-old C57BL/6 female mice cultured **a** with indicated doses of NR for 5 days and **b** with 1 mM NR for the indicated time period (triplicates). AFU arbitrary fluorescence units. **c** Protein by western blot and **d** mRNA by qRT-PCR (triplicates) in cells cultured with 1 mM NR as in **a**. **e** Representative images (left) and quantification (right) of Alizarin Red staining of bone marrow stromal cells from 24-month-old C57BL/6 female mice cultured under osteogenic conditions for 21 days (triplicates) with or without 1 mM of NR. The data presented are mean ± SD using pooled cells from five mice analyzed by one-way ANOVA (**a**, **b**) or Student’s *t*-test (**c**, **e**).

To determine whether the decline in NAD^+^ could contribute to the decreased osteoblastogenesis with aging, we cultured bone marrow-derived stromal cells from 24-month-old C57BL/6 mice in the presence of NR. As anticipated, NR increased NAD^+^ levels in the cultured cells (Fig. 2a, b). Moreover, NR decreased acetylation of both FoxO1 and β-catenin (Fig. 2c), perhaps due to an increase in Sirt1 activity. As an independent indicator of NAD^+^ levels, we also examined the activity of PARP1. PARPs are activated by DNA damage and catalyze the NAD-dependent polymerization of a chain of polymeric adenosine diphosphate ribose (poly(ADP)-ribose or PAR), which signals repair of DNA^27^. NR increased both PARP1 levels and activity as determined by the synthesis of PAR (Fig. 2c). In addition, NR decreased markers of senescence including p21 and Gata4 protein levels (Fig. 2c). As seen before^13^, expression of p16 was not detected in osteoprogenitors from old mice (data not shown). The mRNA expression of known components of the senescence associated secretory phenotype (SASP) such as *IL-1α*, *Mmp13*, and *Cxcl12* were also decreased by NR (Fig. 2d). In line with these molecular events, NR increased osteoblastogenesis in bone marrow-derived stromal cells from old mice cultured for 21 days in osteogenic medium (Fig. 2e).

### Long-term NAD^+^ supplementation attenuates skeletal aging

**Fig. 3:**
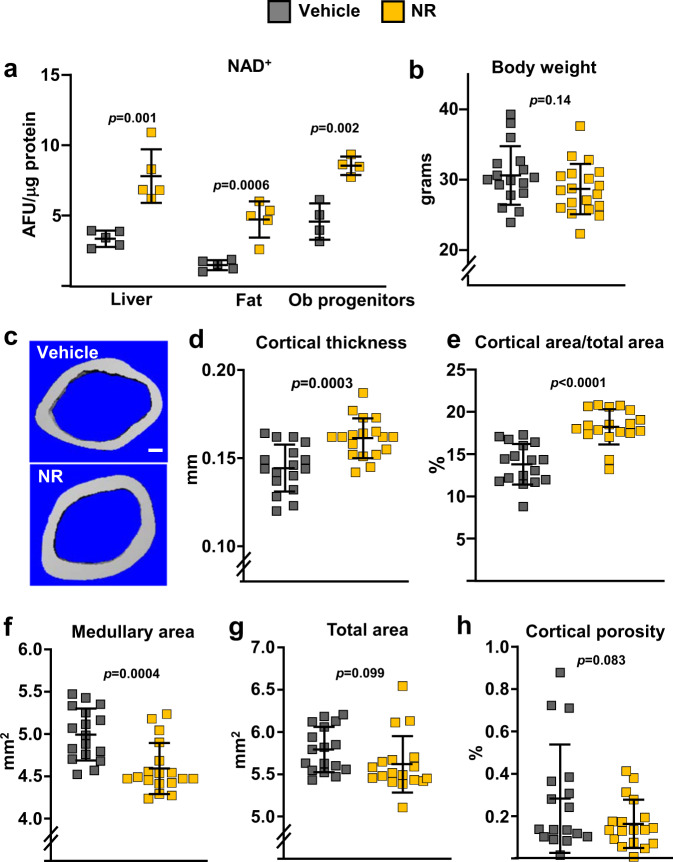
Administration of NR to aging mice attenuates the loss of bone mass. Twelve-month-old C57BL/6 female mice were treated without or with 12 mM NR, administered in the drinking water, until sacrifice at the age of 20 months. **a** NAD^+^ levels in liver, fat tissues, and cultured bone marrow stromal cells (*n* = 4–5/group). **b** Body weight before sacrifice (*n* = 18–19/group). **c** Representative images of cortical bone, **d** cortical thickness, **e** cortical area/total area, **f** medullary area, **g** total area, and **h** cortical porosity by micro‐CT at the distal metaphysis of femur (*n* = 18–19/group). Data represent mean ± SD; *p* values by two-tailed unpaired *t*-test.

To test whether a decline in NAD^+^ mediates skeletal aging, 12-month-old female C57BL/6 mice were administered NR in the drinking water until sacrifice at the age of 20 months. As anticipated, NR treatment resulted in markedly increased NAD^+^ levels in liver, fat tissue, and in bone marrow-derived osteoblastic cells (Fig. 3a). NR-treated mice exhibited no changes in body weight (Fig. 3b). Importantly, NR-treated mice had higher femoral cortical thickness and area than controls, as determined by micro-CT (Fig. 3c–e). This effect was due to a decrease in medullary area (Fig. 3f) while no changes were detected in total area (Fig. 3g). NR had no effect on cortical porosity (Fig. 3h) or on trabecular bone mass and microarchitecture of the spine, including trabecular number, thickness, and separation (Supplementary Fig. 1).

**Fig. 4:**
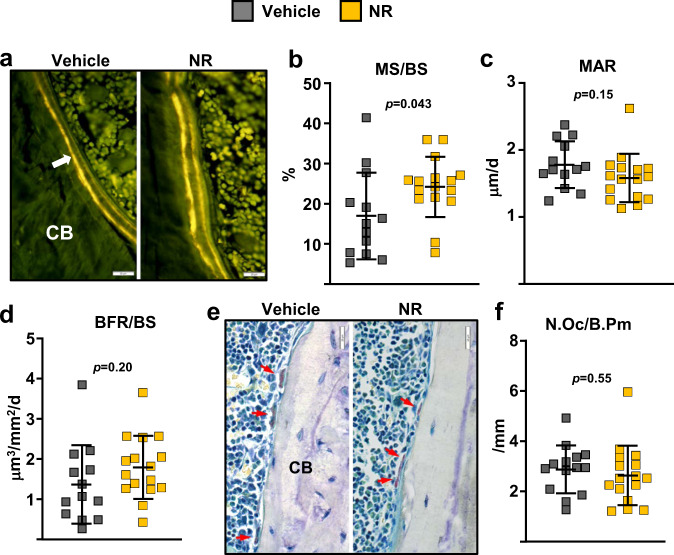
Mice administered with NR had higher mineralizing surface. **a** Representative photomicrographs of endocortical bone surface labeled with tetracycline (fluorescent green) in longitudinal undecalcified femur sections from 20-month-old female mice treated without or with 12 mM NR (×40 magnification). **b** Mineralizing surface, **c** mineral apposition rate, and **d** bone formation rate determined by tetracycline labels (*n* = 13–16 mice/group, one section per mouse was analyzed). **e** Representative microphotographs of femur sections as in **a** stained with TRAP. Red arrows indicate osteoclasts (×40 magnification). **f** Osteoclast number per bone perimeter determined in sections stained with TRAP (*n* = 13–16 mice/group, one section per mouse was analyzed). Data represent mean ± SD; *p* values by two-tailed unpaired *t*-test.

We next searched for the cellular mechanisms responsible for the effects of the NR by performing histomorphometric analysis at the endocortical surface of the femur. Mineralization surface, an indicator of osteoblast number, was increased by 43% in NR-treated mice (Fig. 4a, b). In contrast, mineral apposition rate—a measurement of the vigor of individual osteoblasts—was not affected by NR (Fig. 4c). Nonetheless, the changes in mineralization surface alone were not sufficient to alter bone formation rate (BFR), a measure calculated by multiplying mineralizing surfaces by mineral apposition rate (Fig. 4d). The number of osteoclasts was unaffected by NR (Fig. 4e, f).

### Administration of NR attenuates the decline in osteoprogenitors with aging

**Fig. 5:**
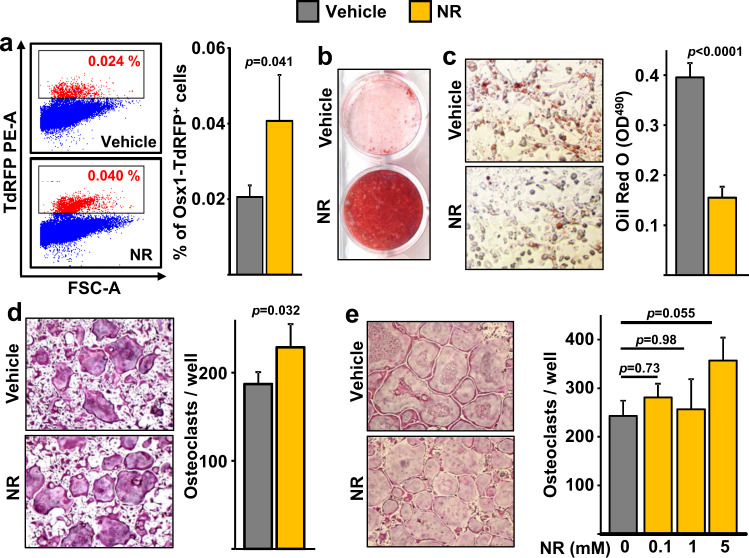
Administration of NR to aging mice increases osteoprogenitor number. **a** Representative flow cytometry analysis (left) and percentage (right) of Osx1-TdRFP^+^ cells in the bone marrow of 20-month-old Osx1-Cre;TdRFP mice treated without or with 12 mM NR for 8 months (*n* = 4 mice/group). **b** Representative images of Alizarin Red staining of bone marrow stromal cells, from mice described in Fig. 3, cultured under osteogenic conditions for 21 days. **c** Representative images (left) and quantification (right) of Oil Red O Staining in bone marrow stromal cells, from mice described in Fig. 3, cultured under adipogenic conditions for 10 days (triplicate cultures). **d**, **e** Representative images (right) and quantification (left) of TRAP-positive osteoclasts containing three or more nuclei in cultures of bone marrow macrophages with M-CSF and RANKL for 5 days, from mice described in Fig. 3 (quadruplicate cultures) (**d**); or from untreated 24-month-old C57BL/6 female mice in the presence of the indicated doses of NR (quadruplicate cultures) (**e**). Data represent mean ± SD; *p* values by two-tailed *t*-test (**a**, **c**, **d**) or one-way ANOVA (**e**).

In an attempt to explain the improvements in mineralizing surface with NAD^+^ repletion, we examined effects in osteoprogenitors. The transcription factor Osx1 is required for the differentiation of mesenchymal progenitors into osteoblasts^28^. Using mice in which osteoprogenitor cells expressing *Osx1-Cre* are labeled with a red fluorescent protein (Osx1-Cre;TdRFP mice), we have previously shown that the number of Osx1-TdRFP^+^ cells greatly declines with aging, and that bone marrow stromal cells from old mice form fewer mineralized nodules when cultured under osteogenic conditions^13^. Female Osx1-Cre;TdRFP mice were administered vehicle or NR using the same experimental protocol described above for wild-type C57BL/6 mice. The number of Osx1-TdRFP^+^ cells in 20-month-old mice treated with NR was approximately twofold higher than in controls (Fig. 5a). Consistent with these results, bone marrow stromal cell cultures from NR-treated mice showed increased osteoblast formation when cultured under osteogenic conditions, as determined by Alizarin Red staining of mineralized matrix (Fig. 5b). In contrast, the number of Oil Red O-positive cells, formed in cultures of stromal cells treated with the PPARγ stimulator rosiglitazone, was much lower in cultures from mice receiving NR (Fig. 5c). To examine whether NR could alter the osteoclastogenic potential of hematopoietic cells, bone marrow-derived macrophages (BMMs) from the mice treated with NR or with vehicle were cultured in the presence of macrophage colony-stimulating factor (M-CSF) and RANKL. Osteoclast formation was slightly increased in cultures from mice treated with NR (Fig. 5d). Likewise, addition of 5 mM of NR to BMM cultures from 24-month-old C57BL/6 mice increased osteoclast formation, while lower doses had no effect (Fig. 5e). These results indicate that NR can increase osteoclast formation in cultured cells. Yet, NR administration to mice was not sufficient to alter osteoclast number in bone (Fig. 4f). Overall, these findings support the premise that the beneficial effects of NR in aging bone result from actions in cells of the osteoblast lineage.

### Low NAD^+^ levels inhibit osteoblastogenesis at least in part via FoxOs

**Fig. 6:**
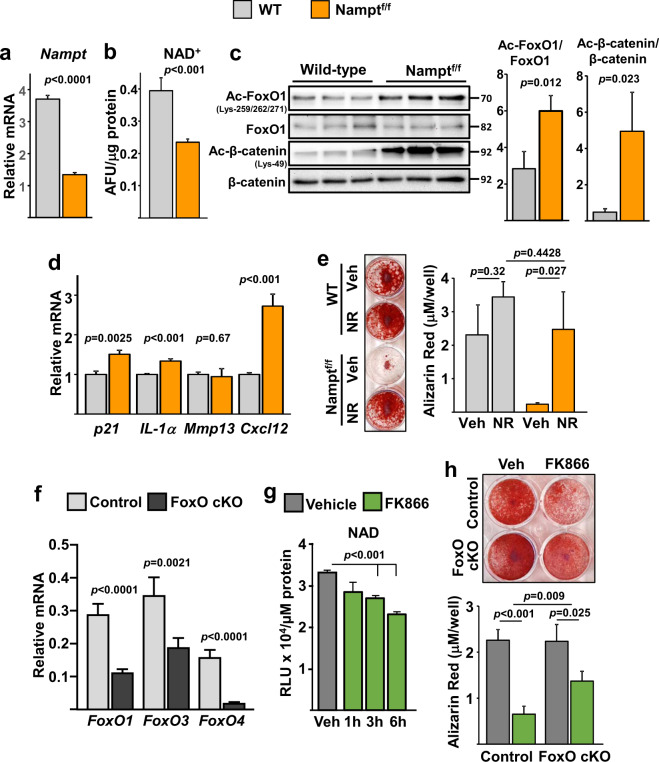
Inhibition of Nampt promotes FoxO and β-catenin acetylation and decreases osteoblastogenesis. **a**–**e** Bone marrow stromal cells from WT or Nampt^f/f^ mice, infected with Ad-Cre and cultured under osteogenic conditions for 5 days. **a** mRNA expression by qRT-PCR. **b** Relative cellular NAD^+^ levels. AFU arbitrary fluorescence units. **c** Protein levels by western blot. Each band represents one mouse. **d** mRNA expression by qRT-PCR. **e** Representative images (left) and quantification of Alizarin Red staining (right) of bone marrow stromal cells, described in **a**, cultured under osteogenic conditions for 21 days. **f** mRNA expression in bone marrow stromal cells from 3-month-old control (FoxO1,3,4^f/f^) or cKO (FoxO1,3,4^ΔOsx1-Cre^) littermate mice cultured under osteogenic conditions for 5 days. **g** Time-dependent effects of 10 µM FK866 on NAD levels in bone marrow cells from 6-month-old C57BL/6 female mice. **h** Representative images (top) and quantification of Alizarin Red staining (bottom) of bone marrow stromal cells described in **f**, cultured under osteogenic conditions without or with 10 µM FK866 for 21 days. In all experiments except **c**, data were obtained from triplicate cultures from cells pooled from three mice per group. Data represent mean ± SD; *p* values by two-tailed *t*-test (**a**–**d**, **f**), one-way ANOVA (**g**), or two-way ANOVA (**h**).

To further examine the role of NAD^+^ in osteoblastic cells, we inhibited the NAD salvage pathway. To this end, we cultured bone marrow stromal cells from mice with floxed alleles for *Nampt* (*Nampt*^fl^)^29^ and deleted *Nampt* using adeno-Cre (Fig. 6a). As expected, ablation of *Nampt* decreased the levels of NAD^+^ (Fig. 6b). This was associated with increased acetylation of FoxO1 and β-catenin (Fig. 6c), re-enforcing the notion that the acetylation status of these proteins is highly dependent on NAD^+^ levels. In addition, cells lacking *Nampt* had increased mRNA expression of *p21* and elements of the SASP such as *IL-1α* and *Cxcl12* (Fig. 6d). Nampt deletion greatly decreased osteoblastogenesis and addition of NR to the cultures prevented this effect (Fig. 6e). To examine the contribution of FoxOs to the deleterious effects of NAD depletion on osteoblastogenesis we used bone marrow-derived stromal cell cultures from control or mice with conditional deletion of FoxOs in osteoblastic cells. As previously shown^26^, the mRNA levels of *FoxO1, 3*, and *4* was greatly decreased in these cultures (Fig. 6f). The Nampt inhibitor FK866 was used to lower NAD levels (Fig. 6g). FK866 decreased osteoblastogenesis in cells from control mice but this effect was greatly attenuated in cells lacking FoxOs (Fig. 6h).

### Deletion of Nampt in mesenchymal progenitors decreases bone mass in young mice

**Fig. 7:**
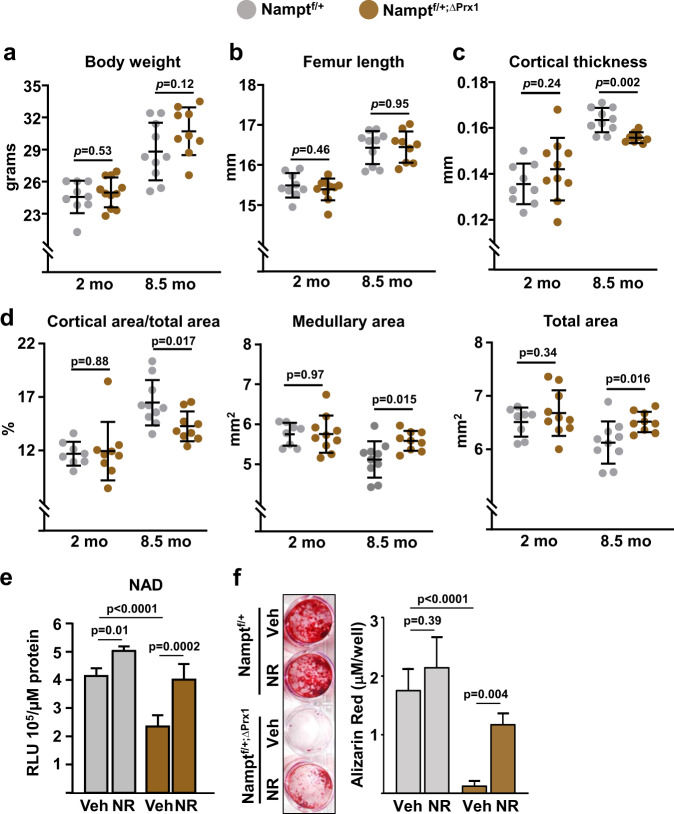
Deletion of *Nampt* in mesenchymal stem cells accelerates skeletal aging. Male Nampt^f/+;∆Prx1^ and Nampt^f/+^ control littermates were generated and sacrificed at 2 (*n* = 10 and 9, respectively) and 8.5 months of age (*n* = 9 and 10, respectively). **a** Body weight measured immediately before sacrifice. **b** Length measured with calipers in dissected femurs. **c** Cortical thickness and **d** cortical areas at the distal metaphysis of femur by micro-CT. **e** Cellular NAD^+^ levels in bone marrow stromal cell cultures from 8.5-month-old mice, in the presence or absence of 1 mM NR (quadruplicate). RLU relative luminescence units. **f** Representative images (left) and quantification (right) of Alizarin Red staining in bone marrow stromal from 8.5-month-old mice cultured under osteogenic conditions for 21 days, in the presence or absence of 1 mM NR (triplicates). Data represent mean ± SD; *p* values by two-way ANOVA.

To determine the skeletal effects of NAD^+^ attenuation specifically in mesenchymal cells, we crossed Nampt^fl^ with Prx1-Cre mice to cause *Nampt* deletion in mesenchymal stem cells, chondrocytes, osteoblast progenitors, osteoblasts, and osteocytes of the appendicular skeleton. Because homozygous deletion of *Nampt* caused severe developmental defects, we used mice with heterozygous deletion (Nampt^fl/+;ΔPrx1^) in our experiments. Nampt^fl/+;ΔPrx1^ mice were born at Mendelian ratios and exhibited similar body weight and femoral length at 2 and 8.5 months of age (Fig. 7a, b), indicating the absence of growth defects. *Nampt* deletion had no effect on trabecular bone mass or microarchitecture at any age (Supplementary Fig. 2). At 2 months of age, cortical thickness and areas were also indistinguishable between mice of the two genotypes (Fig. 7c, d). Between 2 and 8.5 months femoral cortical thickness and area increased and medullary area decreased in Namp^fl/+^ control mice (Fig. 7c, d). At 8.5 months, Nampt^fl/+;ΔPrx1^ mice had lower cortical thickness and area than control mice due to larger medullary area (Fig. 7c, d). Total bone area was also higher in Nampt^fl/+;ΔPrx1^ mice at this age; however, this change was not sufficient to compensate for the larger medullary cavity (Fig. 7d). Cultured bone marrow stromal cells from Nampt^fl/+;ΔPrx1^ mice exhibited higher acetylation of FoxO1 and β-catenin, as well as increased *p21* protein levels (Supplementary Fig. 3). As anticipated, cells from Nampt^fl/+;ΔPrx1^ mice exhibited lower NAD^+^ levels (Fig. 7e) and impaired mineralization when cultured under osteogenic conditions (Fig. 7f). Addition of NR to the cultures normalized NAD^+^ levels and greatly improved mineralization confirming that the defects seen in cells from Nampt^fl/+;ΔPrx1^ mice were due to a deficiency in NAD.

## Discussion

Here we show that NAD^+^ supplementation by the NAD^+^ precursor NR can restore a youthful number of osteoprogenitor cells and attenuate skeletal aging in female mice. These, along with the findings that the levels of NAD^+^ decline with age in osteoblast progenitors, strongly suggest that NAD^+^ is a major target of aging in osteoblastic cells. A decrease in NAD^+^ was also seen in bone marrow stromal cells from 15-month-old when compared to 1-month-old mice^30^. In agreement with our findings, long-term administration of NMN increased bone mineral density in male C57BL/6 mice^31^. In contrast, administration of NMN to 12-month-old mice for only 3 months was not sufficient to alter bone mass^32^.

The decrease in NAD^+^ with age in osteoblast progenitors was associated with an increase in Cd38—the main nicotinamide nucleotidase in mammalian tissues^33^. Cd38 is a multifunctional protein involved in the generation of the second messengers ADPR and cyclic-ADPR (cADPR) which promote intracellular calcium signaling^34^. Due to its NADase activity Cd38 is also a major contributor to cellular and tissue NAD^+^ homeostasis^35^. Similar to our findings in osteoblast progenitors, the levels and activity of Cd38 increase with aging in liver, adipose tissue, spleen, and skeletal muscle^36^. Importantly, genetic or pharmacology inhibition of Cd38 in mice increases NAD^+^ levels in multiple organs and prevents the age-related NAD^+^ decline, attenuates mitochondrial dysfunction, and improves glucose tolerance, cardiac function, and exercise capacity^36–39^. In multiple cell types, inflammatory cytokines such as TNF promote Cd38 expression via activation of NF-kB^40,41^. These, along with the findings that the expression of inflammatory cytokines increases with age in multiple bone cell populations^13,42^ and that NF-kB is stimulated in osteoprogenitors from aged mice^13^, represent a potential explanation for the age-associated increase of Cd38 in osteoblast progenitors.

We also found that the protein levels of Nampt in osteoblastic cells from old mice were lower than in cells from young mice. These along with the findings that deletion of Nampt in mesenchymal lineage cells is sufficient to decrease bone mass support the premise that the age-associated decrease in NAD^+^ in osteoblast progenitors attenuates bone formation. Further support is provided by evidence that NR administration increases osteoprogenitor number and mineralizing surface in aging mice. In tissues such as muscle and intestine, progenitor cells are critical targets of the anti-aging effects of NR^43,44^. Nonetheless, the systemic nature of NR treatment precludes definitive conclusion about the target cells responsible for the beneficial effects on the skeleton.

We and others have shown that osteoprogenitors from old humans or mice exhibit markers of cellular senescence^13,42,45^. Elimination of senescent cells via genetic or pharmacologic manipulations increases bone mass in aged mice, suggesting that cellular senescence contributes to skeletal aging^46^. Our present findings that NR administration decreases markers of senescence in osteoblast progenitors from old mice provide strong support for the contention that a decline in NAD^+^ is a major contributor to the age-associated bone cell senescence. This contention is further supported by evidence that a decrease in NAD^+^ exacerbates replicative senescence in bone marrow-derived stromal cell cultures^47^. NR administration also attenuates cellular senescence in brain and skin of aged mice^43^. Interestingly, in macrophages and endothelial cells Cd38 expression can be induced by factors associated with the SASP^48^, suggesting that cellular senescence re-enforces the decline in NAD^+^.

In most tissues, the downstream mechanisms mediating the beneficial effects of NMN and NR remain unclear. Here, we found that NR decreased the age-related acetylation of FoxOs and β-catenin. Furthermore, lowering NAD^+^ levels via pharmacological or genetic means strongly enhanced acetylation of these proteins. Acetylation of FoxOs increases their association with β-catenin and inhibits Wnt signaling and osteoblastogenesis^18,26^. Sirt1 deacetylates FoxOs and β-catenin and promotes osteoblastogenesis^18,19^. Our findings that osteoblastic cells from mice lacking FoxO1, 3, and 4 are partially protected from the effects of FK866 support the premise that Sirt1/FoxOs mediate the effects of NAD+ on osteoblastogenesis. Further support is provided by evidence that administration of Sirt1 stimulators to mice attenuates skeletal aging^20,21^, and that Sirt1 mediates some of the beneficial effects of NR in the liver, muscle, and gut^43,44,49^. Interestingly, both Sirt1 and FoxOs have been linked to cellular senescence^43,50^. Therefore, it is possible that a decline in NAD^+^ with age contributes to osteoprogenitor senescence via Sirt1- and FoxO-dependent mechanisms. However, given the wide range of NAD^+^ targets and the complex interactions between NAD^+^-dependent processes, further work will be required to define the cellular and molecular targets of NAD^+^ in bone.

Our findings that heterozygous deletion of *Nampt* had no impact on bone development and growth but caused loss of bone mass in young adult mice suggest that a decrease in NAD^+^ in mesenchymal lineage cells caused accelerated skeletal aging. In C57BL/6 mice, femoral growth ceases at 6–7 months, and at about 12 months, which is roughly equivalent to 40 years in humans^51^, the marrow space begins to expand and additional bone is slowly added to the periosteum (outer bone surface); however, the former exceeds the latter, leading to a thinner and more fragile cortex. Interestingly, *Nampt* deletion replicated the effects of aging on cortical bone characterized by cortical thinning associated with the expansion of both medullary and total area. The apposition of bone in the periosteum that occurs with advancing age might be compensatory response to the enlargement of medullary cavity in an effort to maintain bone strength^52^. Whether this is the case in Nampt^fl/+;ΔPrx1^ mice requires future studies.

Based on the results of the present work, we propose that intrinsic defects in osteoblast progenitors that cause a decrease in NAD^+^ contribute to the age-related decline in bone formation and bone mass. Repletion of NAD^+^ with precursors such as NR, therefore, may represent a therapeutic approach to age-associated osteoporosis as it does for other age-related pathologies^16,53,54^.

## Methods

### Animal experimentation

C57BL/6J (B6) mice were obtained from the NIA-supported colony at Harlan or purchased from Jackson Laboratory. Mice expressing RFP in osteoblast progenitors *(Osx1-Cre;TdRFP)* were generated by crossing Osx1-Cre^55^ with mice heterozygous or homozygous for an stop-loxP-tdTRFP allele^56^, as described before^13^, and aged up to 12 months. Mice were assigned to vehicle or NR groups by randomization based on body weight and treated without or with 12 mM NR (ChromaDex) administered in the drinking water. The NR water solution was filtered, provided ad libitum in light-protected bottles, and replaced every 2–3 days for 8 months. Mice were maintained on Teklab global 14% protein rodent maintenance diet (Envigo, catalog 2014) containing 14% protein and 4% fat to prevent excessive weight gain. Mice with tumors were excluded from the experiment. To allow for quantification of BFRs, mice were injected with tetracycline (15 mg/kg body weight) 7 and 3 days before euthanasia at 20 months of age.

Mice with conditional deletion of *Nampt* in the mesenchymal lineage were generated by a two-step breeding strategy. Hemizygous *Prx1-cre* transgenic mice (B6.Cg-Tg(*Prrx1-cre*)1Cjt/J; Jackson Laboratories, stock # 5584) were crossed with *Nampt* floxed (^f/f^) mice (C57BL/6 genetic background) (provided by Shin-ichiro Imai, Washington University School of Medicine) to generate mice heterozygous for the *Nampt* floxed allele with and without the *Cre* allele, *Nampt*^*f/+;∆Prx1*^ and *Nampt*^*f/+*^, respectively. These mice were intercrossed to generate Nampt^f/f^ and Nampt^ΔPrx1^ mice. Offspring were genotyped by PCR using the following primer sequences: *Nampt*-flox primer #1 5′TTC CAG GCT ATT CTG TTC CAG 3′ and *Nampt*-flox primer #2 5′ TCT GGC TCT GTG TAC TGC TGA 3′. Mice with homozygous deletion for Nampt in Prx1-Cre-expressing cells were born with severe developmental defects and were not viable. Offspring from all genotypes were tail-clipped for DNA extraction at the time of weaning (21 days) and then group-housed with same sex littermates. Male Nampt^*f/+;∆Prx1*^ were used as experimental mice and Nampt^*f/+*^ littermates were used as controls. The FoxO1,3,4^ΔOsx1-Cre^ and FoxO1,3,4^f/f^ control littermates were generated by crossing FoxO1,3,4^f/f^ mice (mixture of FVBn and 129Sv) with hemizygous Osx1-cre transgenic mice using a two-step breeding strategy described previously^26^. Mice were maintained with a constant temperature of 23 °C, a 12-h light/dark cycle, and had access to food and water ad libitum. Body weight measurements were performed before euthanasia. Investigators were blinded during animal handling and endpoint measurements. Animal use protocols were approved by the UAMS institutional animal care and use committee.

### NAD^+^assay

NAD^+^ was measured in snap-frozen liver, fat tissue, and bone marrow-derived stromal cell lysates from aged mice using the EnzyChrom^TM^ NAD^+^/NADH Assay Kit, according to the manufacturer’s protocol (Bioassay Systems). Bone marrow stromal cells from Nampt^*f/+*^, Nampt^*f/+; ∆Prx1*^, or wild-type C57BL/6 mice were plated in a 96-well white-wall tissue plate (1.5 × 10^4^ cells/ml) in medium supplemented with ascorbic acid (50 µg/ml) for 5 days. NAD^+^ levels were measured with NAD^+^/NADH-Glo reagent (G9071 Promega) following the manufactured instructions and luminescence quantified with a Packard LumiCount^TM^. Protein levels were determined using the Bio-Rad colorimetric assay.

### Micro-CT

Bone architecture was determined on dissected femora and lumbar vertebra (L5) cleaned of adherent tissue. Bones were fixed in Millonig’s phosphate buffer (Leica Microsystems), and stored in 100% ethanol. A Micro-CT40 (Scanco Medical, Brüttiselen, Switzerland) was used to scan and image vertebral and femoral bone and analysis was performed as described previously^57^. Specifically, scans were performed at medium resolution (12 µm isotropic voxel size) for quantitative determinations and integrated into 3-D voxel images (1024 × 1024 pixel matrices for each individual planar stack). A Gaussian filter (sigma = 0.8, support = 1) was applied to all analyzed scans. Key parameters were X-ray tube potential = 55 kVp, X-ray intensity = 145 µA, integration time = 200 ms, and threshold = 200 mg/cm^3^. Trabecular and cortical bone mass and microarchitecture of the femur were determined at the metaphysis. Trabecular parameters were obtained starting 8–10 slices away from the growth plate so as to avoid the growth plate, and proceeding proximally for 151 slices, to obtain cross-sectional images drawn to exclude cortical elements. To obtain cortical bone parameters, only the proximal third (50 slices) of the metaphysis was used for analysis. Cortical bone was measured at a threshold of 200 mg/cm^3^. For cortical porosity measurements, slices were analyzed from a point immediately distal to the third trochanter to the primary spongiosa. After defining endosteal and periosteal boundaries, an additional image processing script (“peel‐iter =2”) was used to eliminate false voids caused by imperfect wrap of the contours to the bone surface. Images were binarized with a threshold of 365 mg/cm^3^, and overall porosity determined with the “cl_image” script to obtain bone volume and void volume. To avoid inclusion of osteocyte lacunae and canalicular space, void volumes <31,104 µm^3^ (18 voxels) were excluded in the determination of porosity.

The fifth lumbar vertebra (L5) was scanned from the rostral growth plate to the caudal growth plate to obtain 233 slices. BV/TV in the vertebra was determined using 100 slices (1.2 mm) of the anterior (ventral) vertebral body immediately inferior (caudal) to the superior (cranial) growth plate. Trabecular bone analyses were performed on contours of cross-sectional images, drawn to exclude cortical bone, as described for femoral trabecular bone.

### Bone histology and histomorphometry

Freshly dissected femurs were fixed for 24 h in 10% Millonig’s formalin, transferred to ethanol, and embedded undecalcified in methyl methacrylate. For histomorphometric measurements, 5-μm-thick longitudinal sections were cut in the medial-lateral plane. Five sections were obtained from each bone. Sections were left unstained for determination of fluorescent tetracycline labeling or stained for tartrate-resistant acid phosphatase (TRAP) using Leukocyte Acid Phosphatase assay kit (Sigma-Aldrich) to enumerate osteoclasts. Histomorphometric examination of bone sections was performed on both endocortical surfaces of the femur with the OsteoMeasure Analysis System (OsteoMetrics, Inc., Decatur, GA, USA), as previously described. All measurements were made in a blinded fashion. To quantify dynamic indexes of bone formation based on tetracycline labeling, total perimeter (B.Pm), single label perimeter (sL.Pm), double label perimeter (dL.Pm), and mineral apposition rate (MAR) were measured. These values were used to calculate mineralizing surface (MS/BS = [1/2 sL.Pm + dL.Pm]/B.Pm × 100;%) and bone formation rate (BFR/BS = MAR × MS/BS; μm^2^/μm/day). The terminology used is recommended by the Histomorphometry Nomenclature Committee of the American Society for Bone and Mineral Research^58^.

### Quantification of bone marrow Osx1-TdRFP^+^cells

Osx1-TdRFP^+^ cells from mouse bone marrow were isolated as described before^13^. Briefly, bone marrow cells were flushed from tibiae and femora into ice-cold FACS buffer and red blood cells removed with RBC lysis buffer (BD Bioscience). Cells were then incubated with biotin-conjugated rat antibodies specific for mouse CD45 (eBioscience, 14-0451, 1:100) and hematopoietic cells depleted with anti-rat IgG Dynabeads (Invitrogen). CD45^-^ Osx1-TdRFP^+^ cells were sorted in an Aria II cell sorter (BD Bioscience) using the PE-A fluorochrome gate.

### Osteoblast/adipocyte differentiation

Total bone marrow cells were obtained by flushing the tibiae and femora. Cells from 4–5 mice of each group were pooled and cultured with 20% FBS, 1% PSG, and 50 μg/ml ascorbic acid (Sigma) in 10-cm culture dishes for 7 days. The medium was replaced every 3 days. Adherent (stromal) cells were re-plated in 12-well tissue culture plates at 0.2 × 10^6^ cells per well with 10% FBS, 1% PSG, 50 μg/ml of ascorbic acid and 10 mM β-glycerophosphate (Sigma) for 5 days, to perform qPCR assays and western blotting, or 21 days to assess mineralization. Mineralized matrix was stained with 40 mM Alizarin Red solution, following the manufacturer’s instructions (Sigma). Alizarin red was quantified after extraction with 10 mM sodium phosphate, 10% cetylpyridinium chloride, pH 7, and absorbance determined at 562 nm. To analyze adipogenic potential, bone marrow-derived stromal cells were cultured to 80% confluence, and the media supplemented with rosiglitazone (5 nM/ml). Medium was changed every 3 days. After 10 days, cells were fixed in 10% formalin in PBS, rinsed, and stained for 30 min with 0.15% Oil Red O (Sigma) in a 55:45 mix of isopropanol and water. Cells were counterstained with 0.5% methyl green (Fisher Scientific) in 0.1 M sodium acetate, pH 4. Oil Red O staining was quantified after extraction of the dye with 1 ml isopropanol and absorbance determined at 490 nm. For all assays cells were plated in triplicate.

### Osteoclast differentiation

Total bone marrow cells were harvested as described above. After red blood cells were removed using ACK buffer (0.01 mM EDTA, 0.011 M KHCO_3_, and 0.155 M NH_4_Cl, pH 7.3), the remaining cells were cultured with 10% FBS, 1% PSG, and 10 ng/ml M-CSF (R&D Systems) for 24 h. Non-adherent cells were cultured in Petri dishes, with 30 ng/ml M-CSF, for 4–5 days to generate BMMs. BMMs were cultured with 30 ng/ml of M-CSF and 30 ng/ml of RANKL (R&D Systems) for 4–5 days to generate osteoclasts. To enumerate osteoclasts, cells were fixed with 10% neutral buffered formalin for 10 min and stained for tartrate-resistant acid phosphatase (TRAP), using the Leukocyte Acid Phosphatase Assay Kit, following the manufacturer’s instructions (Sigma-Aldrich). An osteoclast was defined as a multinuclear (more than 3 nuclei) TRAP-positive cell.

### Adenovirus infection

Bone marrow stromal cells from Nampt^f/f^ mice or wild-type littermates were incubated with α-MEM complete media containing recombinant adenoviruses encoding Cre recombinase (Ad-Cre;Vector Biolabs) at a multiplicity of infection of 100 for 24 h. Cells were then washed twice with α-MEM and re-plated for NAD^+^, mRNA, western blot, and mineralization assays.

### Western blot analysis

Protein was extracted from cultured cells with a buffer containing 20 mM Tris-HCl, 150 mM NaCl, 1% Triton X-100, protease inhibitor mixture, and phosphatase inhibitor cocktail (Sigma-Aldrich) on ice for 30 min and then kept at −80 °C freezer. Protein concentration was determined using the DC Protein Assay Kit (Bio-Rad). The extracted protein (20–40 μg per sample) was subjected to 8–12% SDS-PAGE gels and transferred electrophoretically onto PVDF membranes. The membranes were blocked in 5% non-fat milk/Tris-buffered saline for 2 h and incubated with each primary antibody followed by secondary antibodies conjugated with horseradish peroxidase. Acetylated FoxO1 was detected using a polyclonal antibody recognizing Lys-259, Lys-262, and Lys-271 (Santa Cruz Biotechnology, sc-49437, 1:500), and acetylated β-Catenin with a monoclonal antibody recognizing Lys-49 (Cell Signaling, #9030, 1:1000). The following antibodies were also used to detect their corresponding protein levels: rabbit monoclonal antibodies against FoxO1 (Cell Signaling, #2880, 1:1000), Nampt (Abcam, ab236874, 1:1000), and Cd38 (Abcam, ab216343, 1:1000); rabbit polyclonal antibodies for full-length Parp1 (Cell Signalling, #9542, 1:1000) and β-catenin (Cell Signalling, #9562, 1:1000); mouse monoclonal antibodies against PAR (Enzo Life Sciences, ALX-804-220-R100, 1:1000), Sirt1 (Cell Signalling, #8469, 1:1000), β-actin (Santa Cruz Biotechnology, sc-81178, 1:2000), p21 (Santa Cruz Biotechnology, sc-6246, 1:500), and goat polyclonal antibody for GATA4 (Santa Cruz Biotechnology, sc-1237, 1:500). The membranes were subjected to western blot analysis with ECL reagents (Millipore). Quantification of the intensity of the bands in the autoradiograms was performed using a VersaDocTM imaging system (Bio-Rad). All blots in each figures were derived from the same experiment and they were processed in parallel.

### Quantitative (q)RT-PCR

Total RNA from cell cultures was extracted with TRIzol reagent (Invitrogen) and reverse-transcribed using the High-Capacity cDNA Archive Kit (Applied Biosystems) according to the manufacturer’s instructions. TaqMan quantitative real-time PCR was performed using the following primers (Applied Biosystems): *Nampt* (Mm00451938_m1); *Cd38* (Mm01220906_m1); *TNF-a* (Mm00443258_m1); *Mmp-13* (Mm00439491_m1); *Il-1α* (Mm99999060_m1); *Cxcl12* (Mm00445553_m1); *FoxO1* (Mm00490672_m1); *FoxO3* (Mm00490673_m1); *FoxO4* (Mm00840140_g1). Target gene expression was calculated by normalizing to the housekeeping gene ribosomal protein S2, *Mrps2* (Mm00475528_m1) using the ∆Ct method^59^.

### Statistical analysis

The data were analyzed by analysis of variance (ANOVA) or Student’s *t*-test (independent samples, two sided) using GraphPad Prism 8 or 9 from GraphPad Software, after determining that the data were normally distributed and exhibited equivalent variances. In the event that ANOVA justified post hoc comparisons between group means, these were conducted using Tukey’s multiple-comparisons test.

### Reporting summary

Further information on research design is available in the Nature Research Reporting Summary linked to this article.

## Supplementary information

reporting summary

Supplementary figures and legends

## Data Availability

The data that support the findings of this study are available from the corresponding author upon reasonable request.

## References

[1] Roux C (2012). Burden of non-hip, non-vertebral fractures on quality of life in postmenopausal women: the Global Longitudinal study of Osteoporosis in Women (GLOW). Osteoporos. Int..

[2] Zebaze RM (2010). Intracortical remodelling and porosity in the distal radius and post-mortem femurs of women: a cross-sectional study. Lancet.

[3] Bala Y, Zebaze R, Seeman E (2015). Role of cortical bone in bone fragility. Curr. Opin. Rheumatol..

[4] Nicks KM (2012). Relationship of age to bone microstructure independent of areal bone mineral density. J. Bone Min. Res..

[5] Burghardt AJ, Kazakia GJ, Ramachandran S, Link TM, Majumdar S (2010). Age- and gender-related differences in the geometric properties and biomechanical significance of intracortical porosity in the distal radius and tibia. J. Bone Min. Res..

[6] Shanbhogue VV, Brixen K, Hansen S (2016). Age- and sex-related changes in bone microarchitecture and estimated strength: a three-year prospective study using HRpQCT. J. Bone Min. Res..

[7] Parfitt AM (1994). Osteonal and hemi-osteonal remodeling: the spatial and temporal framework for signal traffic in adult human bone. J. Cell. Biochem.

[8] Parfitt, A. M., Kleerekoper, M. & Villanueva, A. R. in *Osteoporosis* (eds Christianson, C. et al.) 301–308 (Osteopress ApS, 1987).

[9] Halloran BP (2002). Changes in bone structure and mass with advancing age in the male C57BL/6J mouse. J. Bone Miner. Res.

[10] Glatt V, Canalis E, Stadmeyer L, Bouxsein ML (2007). Age-related changes in trabecular architecture differ in female and male C57BL/6J mice. J. Bone Min. Res..

[11] Almeida M (2007). Skeletal involution by age-associated oxidative stress and its acceleration by loss of sex steroids. J. Biol. Chem..

[12] Piemontese M (2017). Old age causes de novo intracortical bone remodeling and porosity in mice. JCI Insight.

[13] Kim HN (2017). DNA damage and senescence in osteoprogenitors expressing Osx1 may cause their decrease with age. Aging Cell.

[14] Imai S, Guarente L (2014). NAD+ and sirtuins in aging and disease. Trends Cell Biol..

[15] McReynolds MR, Chellappa K, Baur JA (2020). Age-related NAD(+) decline. Exp. Gerontol..

[16] Yoshino J, Baur JA, Imai SI (2018). NAD(+) intermediates: the biology and therapeutic potential of NMN and NR. Cell Metab..

[17] Rajman L, Chwalek K, Sinclair DA (2018). Therapeutic potential of NAD-boosting molecules: the in vivo evidence. Cell Metab..

[18] Iyer S (2014). Sirtuin1 (Sirt1) promotes cortical bone formation by preventing beta (β)-catenin sequestration by FoxO transcription factors in osteoblast progenitors. J. Biol. Chem..

[19] Simic P (2013). SIRT1 regulates differentiation of mesenchymal stem cells by deacetylating beta-catenin. EMBO Mol. Med.

[20] Merken EM (2014). SRT2104 extends survival of male mice on a standard diet and preserves bone and muscle mass. Aging Cell.

[21] Zainabadi K, Liu CJ, Caldwell ALM, Guarente L (2017). SIRT1 is a positive regulator of in vivo bone mass and a therapeutic target for osteoporosis. PLoS ONE.

[22] Ucer S (2017). The effects of aging and sex steroid deficiency on the murine skeleton are independent and mechanistically distinct. J. Bone Min. Res..

[23] Almeida M, Han L, Martin-Millan M, O’Brien CA, Manolagas SC (2007). Oxidative stress antagonizes Wnt signaling in osteoblast precursors by diverting beta-catenin from T cell factor- to forkhead box O-mediated transcription. J. Biol. Chem..

[24] Hoogeboom D (2008). Interaction of FOXO with beta-catenin inhibits beta-catenin/T cell factor activity. J. Biol. Chem..

[25] Essers MA (2005). Functional interaction between beta-catenin and FOXO in oxidative stress signaling. Science.

[26] Iyer S (2013). FoxOs attenuate bone formation by suppressing Wnt signaling. J. Clin. Invest..

[27] Fang EF (2017). NAD(+) in aging: molecular mechanisms and translational implications. Trends Mol. Med.

[28] Nakashima K (2002). The novel zinc finger-containing transcription factor osterix is required for osteoblast differentiation and bone formation. Cell.

[29] Rongvaux A (2008). Nicotinamide phosphoribosyl transferase/pre-B cell colony-enhancing factor/visfatin is required for lymphocyte development and cellular resistance to genotoxic stress. J. Immunol..

[30] Li Y (2011). Nicotinamide phosphoribosyltransferase (Nampt) affects the lineage fate determination of mesenchymal stem cells: a possible cause for reduced osteogenesis and increased adipogenesis in older individuals. J. Bone Min. Res.

[31] Mills KF (2016). Long-term administration of nicotinamide mononucleotide mitigates age-associated physiological decline in mice. Cell Metab..

[32] Song J (2019). Nicotinamide mononucleotide promotes osteogenesis and reduces adipogenesis by regulating mesenchymal stromal cells via the SIRT1 pathway in aged bone marrow. Cell Death Dis..

[33] Chini EN, Chini CCS, Espindola Netto JM, de Oliveira GC, van Schooten W (2018). The pharmacology of CD38/NADase: an emerging target in cancer and diseases of aging. Trends Pharmacol. Sci..

[34] Malavasi F (2008). Evolution and function of the ADP ribosyl cyclase/CD38 gene family in physiology and pathology. Physiol. Rev..

[35] Hogan KA, Chini CCS, Chini EN (2019). The multi-faceted Ecto-enzyme CD38: roles in immunomodulation, cancer, aging, and metabolic diseases. Front. Immunol..

[36] Camacho-Pereira J (2016). CD38 dictates age-related NAD decline and mitochondrial dysfunction through an SIRT3-dependent mechanism. Cell Metab..

[37] Tarrago MG (2018). A potent and specific CD38 inhibitor ameliorates age-related metabolic dysfunction by reversing tissue NAD(+) decline. Cell Metab..

[38] Aksoy P, White TA, Thompson M, Chini EN (2006). Regulation of intracellular levels of NAD: a novel role for CD38. Biochem. Biophys. Res. Commun..

[39] Young GS, Choleris E, Lund FE, Kirkland JB (2006). Decreased cADPR and increased NAD+ in the Cd38−/− mouse. Biochem. Biophys. Res. Commun..

[40] Tirumurugaan KG (2007). TNF-alpha induced CD38 expression in human airway smooth muscle cells: role of MAP kinases and transcription factors NF-kappaB and AP-1. Am. J. Physiol. Lung Cell. Mol. Physiol..

[41] Iqbal J, Kumar K, Sun L, Zaidi M (2006). Selective upregulation of the ADP-ribosyl cyclases CD38 and CD157 by TNF but not by RANK-L reveals differences in downstream signaling. Am. J. Physiol. Ren. Physiol..

[42] Farr JN (2016). Identification of senescent cells in the bone microenvironment. J. Bone Min. Res..

[43] Zhang H (2016). NAD(+) repletion improves mitochondrial and stem cell function and enhances life span in mice. Science.

[44] Igarashi M (2019). NAD(+) supplementation rejuvenates aged gut adult stem cells. Aging Cell.

[45] Stenderup K, Justesen J, Clausen C, Kassem M (2003). Aging is associated with decreased maximal life span and accelerated senescence of bone marrow stromal cells. Bone.

[46] Farr J (2017). Targeting cellular senescence prevents age-related bone loss in mice. Nat. Med.

[47] Pi C (2019). Nicotinamide phosphoribosyltransferase postpones rat bone marrow mesenchymal stem cell senescence by mediating NAD(+)-Sirt1 signaling. Aging (Albany NY).

[48] Chini C (2019). The NADase CD38 is induced by factors secreted from senescent cells providing a potential link between senescence and age-related cellular NAD(+) decline. Biochem Biophys. Res. Commun..

[49] Gariani K (2016). Eliciting the mitochondrial unfolded protein response by nicotinamide adenine dinucleotide repletion reverses fatty liver disease in mice. Hepatology.

[50] Baar MP (2017). Targeted apoptosis of senescent cells restores tissue homeostasis in response to chemotoxicity and aging. Cell.

[51] Dutta S, Sengupta P (2016). Men and mice: relating their ages. Life Sci..

[52] Seeman E (2003). Periosteal bone formation-a neglected determinant of bone strength. N. Engl. J. Med.

[53] Kane AE, Sinclair DA (2018). Sirtuins and NAD(+) in the fevelopment and treatment of metabolic and cardiovascular diseases. Circ. Res..

[54] Katsyuba E, Auwerx J (2017). Modulating NAD(+) metabolism, from bench to bedside. EMBO J..

[55] Rodda SJ, McMahon AP (2006). Distinct roles for Hedgehog and canonical Wnt signaling in specification, differentiation and maintenance of osteoblast progenitors. Development.

[56] Luche H, Weber O, Nageswara RT, Blum C, Fehling HJ (2007). Faithful activation of an extra-bright red fluorescent protein in “knock-in” Cre-reporter mice ideally suited for lineage tracing studies. Eur. J. Immunol..

[57] Martin-Millan M (2010). The estrogen receptor-alpha in osteoclasts mediates the protective effects of estrogens on cancellous but not cortical bone. Mol. Endocrinol..

[58] Dempster DW (2013). Standardized nomenclature, symbols, and units for bone histomorphometry: a 2012 update of the report of the ASBMR Histomorphometry Nomenclature Committee. J. Bone Min. Res..

[59] Livak KJ, Schmittgen TD (2001). Analysis of relative gene expression data using real-time quantitative PCR and the 2(-Delta Delta C(T)) method. Methods.

