# Supplementary material for: A decrease in NAD+ contributes to the loss of osteoprogenitors and bone mass with aging
Source: NPJ Aging Mech Dis. 2021 Apr 1;7:8. doi: 10.1038/s41514-021-00058-7 (PMC8016898; doi:10.1038/s41514-021-00058-7)
Supplement: Supplementary file 1 — reporting summary [file 41514_2021_58_MOESM1_ESM.pdf]

nature research

Corresponding author(s): Maria Almeida

Last updated by author(s): Jan 11, 2021

Reporting Summary

Nature Research wishes to improve the reproducibility of the work that we publish. This form provides structure for consistency and transparency in reporting. For further information on Nature Research policies, see our Editorial Policies and the Editorial Policy Checklist.

Statistics

For all statistical analyses, confirm that the following items are present in the figure legend, table legend, main text, or Methods section.

n/a	Confirmed	
☐	☒	The exact sample size (*n*) for each experimental group/condition, given as a discrete number and unit of measurement
☐	☒	A statement on whether measurements were taken from distinct samples or whether the same sample was measured repeatedly
☐	☒	The statistical test(s) used AND whether they are one- or two-sided<br>*Only common tests should be described solely by name; describe more complex techniques in the Methods section.*
☐	☒	A description of all covariates tested
☐	☒	A description of any assumptions or corrections, such as tests of normality and adjustment for multiple comparisons
☐	☒	A full description of the statistical parameters including central tendency (e.g. means) or other basic estimates (e.g. regression coefficient) AND variation (e.g. standard deviation) or associated estimates of uncertainty (e.g. confidence intervals)
☐	☒	For null hypothesis testing, the test statistic (e.g. *F*, *t*, *r*) with confidence intervals, effect sizes, degrees of freedom and *P* value noted<br>*Give P values as exact values whenever suitable.*
☒	☐	For Bayesian analysis, information on the choice of priors and Markov chain Monte Carlo settings
☒	☐	For hierarchical and complex designs, identification of the appropriate level for tests and full reporting of outcomes
☒	☐	Estimates of effect sizes (e.g. Cohen's *d*, Pearson's *r*), indicating how they were calculated

Our web collection on statistics for biologists contains articles on many of the points above.

Software and code

Policy information about availability of computer code

Data collection	NAD+/NADH-Glo reagent (G9071 Promega) following the manufactured instructions and luminescence quantified with a Packard LumiCountTM. A Micro-CT40 (Scanco Medical, Brüttiselen, Switzerland) was used to scan and image vertebral and femoral bone. Histomorphometric examination of bone sections was performed on both endocortical surfaces of the femur with the OsteoMeasure Analysis System (OsteoMetrics, Inc., Decatur, GA, USA). CD45- Osx1-TdRFP+ cells were sorted in an Aria II cell sorter (BD Bioscience) using the PE-A fluorochrome gate.
Data analysis	Quantification of the intensity of the bands in the autoradiograms was performed using a VersaDocTM imaging system (Bio-Rad). The data were analyzed by analysis of variance (ANOVA) or Student's t test (independent samples, two-sided) using GraphPad Prism 8 or 9 from GraphPad Software, after determining that the data were normally distributed and exhibited equivalent variances.

For manuscripts utilizing custom algorithms or software that are central to the research but not yet described in published literature, software must be made available to editors and reviewers. We strongly encourage code deposition in a community repository (e.g. GitHub). See the Nature Research guidelines for submitting code & software for further information.

Data

Policy information about availability of data

All manuscripts must include a data availability statement. This statement should provide the following information, where applicable:

- Accession codes, unique identifiers, or web links for publicly available datasets
- A list of figures that have associated raw data
- A description of any restrictions on data availability

The data that support the findings of this study are available from the corresponding author upon reasonable request.

Field-specific reporting

Please select the one below that is the best fit for your research. If you are not sure, read the appropriate sections before making your selection.

- [x] Life sciences
- [ ] Behavioural & social sciences
- [ ] Ecological, evolutionary & environmental sciences

For a reference copy of the document with all sections, see nature.com/documents/nr-reporting-summary-flat.pdf

Life sciences study design

All studies must disclose on these points even when the disclosure is negative.

Sample size	Sample sizes were designed based on prior assay experience, similar work on related projects, and pilot data.
Data exclusions	No data were excluded.
Replication	Adequate measures were taken to verify the reproducibility of findings. An effect was confirmed across multiple mouse cohorts, cell culture conditions with independent experiments and using several biological and technical replicates. The compounds were tested at multiple concentrations in vitro and the in vivo results were confirmed in different model systems such as in naturally aged mice. Some results were confirmed by different investigators and in different laboratory conditions. All attempts at replication were successful.
Randomization	Animals were randomized in different groups on the basis of body weight in a way that each group had nearly equal average body weight at the start of treatment.
Blinding	The investigators were blinded during data collection and analysis.

Reporting for specific materials, systems and methods

We require information from authors about some types of materials, experimental systems and methods used in many studies. Here, indicate whether each material, system or method listed is relevant to your study. If you are not sure if a list item applies to your research, read the appropriate section before selecting a response.

Materials & experimental systems

n/a	Involved in the study	
[ ]	[x]	Antibodies
[x]	[ ]	Eukaryotic cell lines
[x]	[ ]	Palaeontology and archaeology
[ ]	[x]	Animals and other organisms
[x]	[ ]	Human research participants
[x]	[ ]	Clinical data
[x]	[ ]	Dual use research of concern

Methods

n/a	Involved in the study	
[x]	[ ]	ChIP-seq
[ ]	[x]	Flow cytometry
[x]	[ ]	MRI-based neuroimaging

Antibodies

Antibodies used	Acetylated FoxO1 was detected using a polyclonal antibody recognizing Lys-259, Lys-262, and Lys-271 (Santa Cruz Biotechnology, sc-49437, 1:500), and acetylated β-Catenin with a monoclonal antibody recognizing Lys-49 (Cell Signaling, #9030, 1:1000). The following antibodies were also used to detect their corresponding protein levels: rabbit monoclonal antibodies against FoxO1 (Cell Signaling, #2880, 1:1000), Nampt (abcam, ab236874, 1:1,000), and Cd38 (abcam, ab216343, 1:1,000), rabbit polyclonal antibodies for full length Parp1 (Cell Signalling, #9542, 1:1,000) and β-catenin (Cell Signalling, #9562, 1:1,000), mouse monoclonal antibodies against PAR (Enzo Life Sciences, ALX-804-220-R100, 1:1000), Sirt1 (Cell Signalling, #8469, 1:1,000), β-actin (Santa Cruz Biotechnology, sc-81178, 1:2000), p21 (Santa Cruz Biotechnology, sc-6246, 1:500), and goat polyclonal antibody for GATA4 (Santa Cruz Biotechnology, sc-1237, 1:500).
Validation	All the used antibodies are commercially available. The antibodies used in a specific species or application have been validated by manufacturers to be used in that species/application and this information is provided in their website and/or antibody datasheets.

Animals and other organisms

Policy information about studies involving animals; ARRIVE guidelines recommended for reporting animal research

Laboratory animals	C57BL/6J (B6) mice were obtained from the NIA-supported colony at Harlan or purchased from Jackson Laboratory. Mice expressing RFP in osteoblast progenitors (Osx1-Cre;TdRFP) were generated by crossing Osx1-Cre57 with mice heterozygous or homozygous for an stop-loxP-tdTRFP allele58, as described before14, and aged up to 12 months. Mice were assigned to vehicle or NR groups by

randomization based on body weight and treated without or with 12 mM NR (ChromaDex) administered in the drinking water. The NR water solution was filtered, provided ad libitum in light-protected bottles, and replaced every 2-3 days for 8 months. Mice were maintained on Teklab global 14% protein rodent maintenance diet (Envigo, catalog 2014) containing 14% protein and 4% fat to prevent excessive weight gain. Mice with tumors were excluded from the experiment. To allow for quantification of bone formation rates, mice were injected with tetracycline (15mg/kg body weight) 7 and 3 days before euthanasia at 20 months of age.

Mice with conditional deletion of Nampt in the mesenchymal lineage were generated by a two-step breeding strategy. Hemizygous Prx1-cre transgenic mice (B6.Cg-Tg(Prrx1-cre)1Cjt/J; Jackson Laboratories, stock # 5584) were crossed with Nampt floxed (f/f) mice (C57BL/6 genetic background) (provided by Shin-ichiro Imai, Washington University School of Medicine) to generate mice heterozygous for the Nampt floxed allele with and without the Cre allele, Namptf/+;ΔPrx1 and Namptf/+, respectively. These mice were intercrossed to generate Namptf/f and NamptΔPrx1 mice.Mice with homozygous deletion for Nampt in Prx1-Cre expressing cells were born with severe developmental defects and were not viable. Offspring from all genotypes were tail-clipped for DNA extraction at the time of weaning (21 days) and then group-housed with same sex littermates. Male Namptf/+;ΔPrx1 were used as experimental mice and Namptf/+ littermates were used as controls. The FoxO1,3,4 ΔOsx1-Cre and FoxO1,3,4f/f control littermates were generated by crossing FoxO1,3,4f/f mice (mixture of FVBn and 129Sv) with hemizygous Osx1-cre transgenic mice using a two-step breeding strategy described previously. Mice were maintained with a constant temperature of 23°C, a 12-hour light/dark cycle, and had access to food and water ad libitum. Body weight measurements were performed before euthanasia. Investigators were blinded during animal handling and endpoint measurements. Animal use protocols were approved by the UAMS institutional animal care and use committee.

Wild animals	The study did not involve wild animals.
Field-collected samples	The study did not involve samples collected from the field.
Ethics oversight	All animal work was approved and done in accordance with the UAMS institutional animal care and use committee. All animal studies were complied with the ethical regulations and humane endpoint according to the NIH Guidelines for the Care and Use of Laboratory Animals.

Note that full information on the approval of the study protocol must also be provided in the manuscript.

Flow Cytometry

Plots

Confirm that:

- [x] The axis labels state the marker and fluorochrome used (e.g. CD4-FITC).
- [x] The axis scales are clearly visible. Include numbers along axes only for bottom left plot of group (a 'group' is an analysis of identical markers).
- [x] All plots are contour plots with outliers or pseudocolor plots.
- [x] A numerical value for number of cells or percentage (with statistics) is provided.

Methodology

Sample preparation	The tibiae and femurs were dissected from mice immediately after death. Total bone marrow cells were flushed from the bones, using a 23-gauge needle and syringe, into ice-cold FACS buffer containing CaCl2- and MgCl2-free 1X PBS (Thermo Fisher Scientific) and 2% FBS. Cells from individual mice in each group were centrifuged at 1400 rpm for 6 min at 4 °C. After the red blood cells were removed with RBC lysis buffer (0.9 % NH4Cl with 20 mM Tris base, pH 7.4), bone marrow cells were suspended in ice-cold FACS buffer. Cells were then incubated with biotin-conjugated rat antibodies specific for mouse CD45 (eBioscience, 14-0451, 1:100). The labeled hematopoietic cells were depleted 3 times by incubation with anti-rat IgG Dynabeads (Invitrogen) at a bead:cell ratio of approximately 4:1. Cells binding the Dynabeads were removed with a magnetic field. The negatively isolated CD45- cells were washed twice and suspended with ice-cold FACS buffer at 1-2x106 cells/ml. Osx1-TdRFP+ cells were sorted in an Aria II cell sorter (BD Bioscience) using the PE-A fluorochrome gate.
Instrument	Aria II cell sorter (BD Bioscience)
Software	FlowJo, V10
Cell population abundance	Approximately, 100,000 events of live cells were collected.
Gating strategy	FSC-A/SSC-A was used to distinguish the cell size. PI or 7-AAD negative was used to gate the live cells. FSC-A was used to gate the single cells, all single live cells were analyzedTdRFP+ cells were sorted in an Aria II cell sorter (BD Bioscience) using the PE-A fluorochrome gate.

- [x] Tick this box to confirm that a figure exemplifying the gating strategy is provided in the Supplementary Information.
