# Supplementary material for: A decrease in NAD+ contributes to the loss of osteoprogenitors and bone mass with aging
Source: NPJ Aging Mech Dis. 2021 Apr 1;7:8. doi: 10.1038/s41514-021-00058-7 (PMC8016898; doi:10.1038/s41514-021-00058-7)
Supplement: Supplementary file 2 — Supplementary figures and legends [file 41514_2021_58_MOESM2_ESM.pdf]

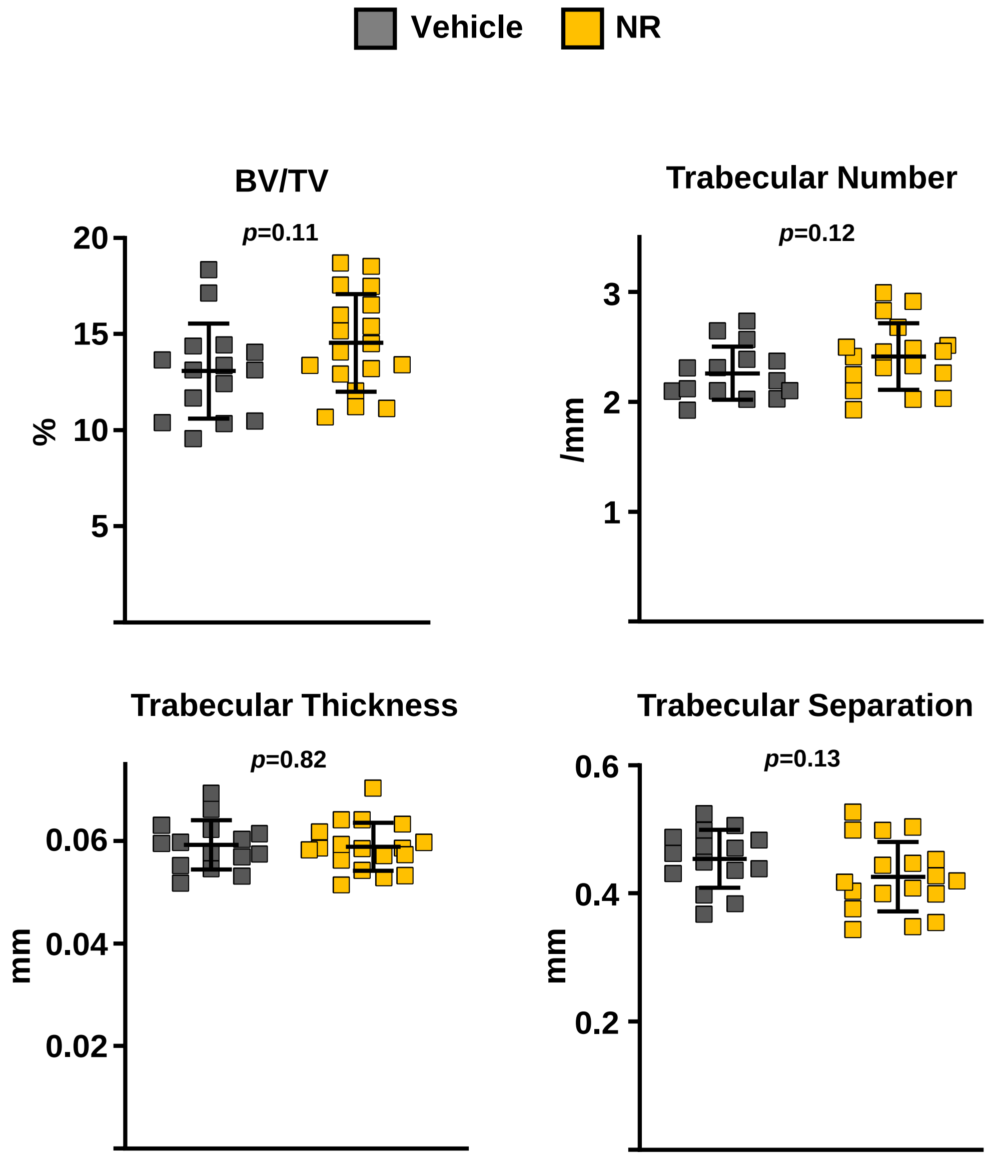

Vehicle
NR
BV/TV
p=0.11
%
20
15
10
5
Trabecular Number
p=0.12
/mm
3
2
1
Trabecular Thickness
p=0.82
mm
0.06
0.04
0.02
Trabecular Separation
p=0.13
mm
0.6
0.4
0.2

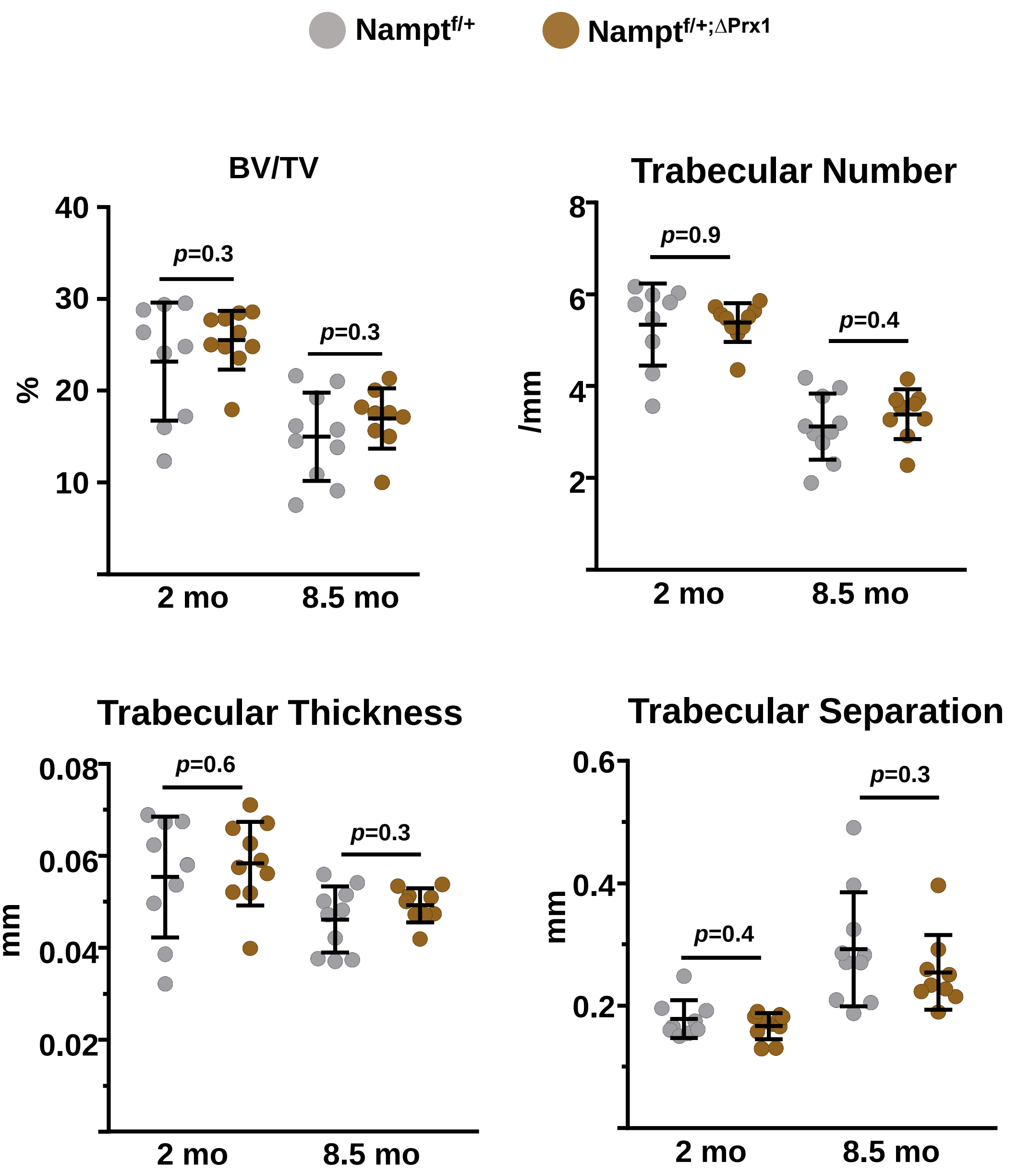

$Nampt^{f/+}$
$Nampt^{f/+;\Delta Prx1}$
BV/TV
%
40
30
20
10
p=0.3
p=0.3
2 mo
8.5 mo
Trabecular Number
/mm
8
6
4
2
p=0.9
p=0.4
2 mo
8.5 mo
Trabecular Thickness
mm
0.08
0.06
0.04
0.02
p=0.6
p=0.3
2 mo
8.5 mo
Trabecular Separation
mm
0.6
0.4
0.2
p=0.4
p=0.3
2 mo
8.5 mo

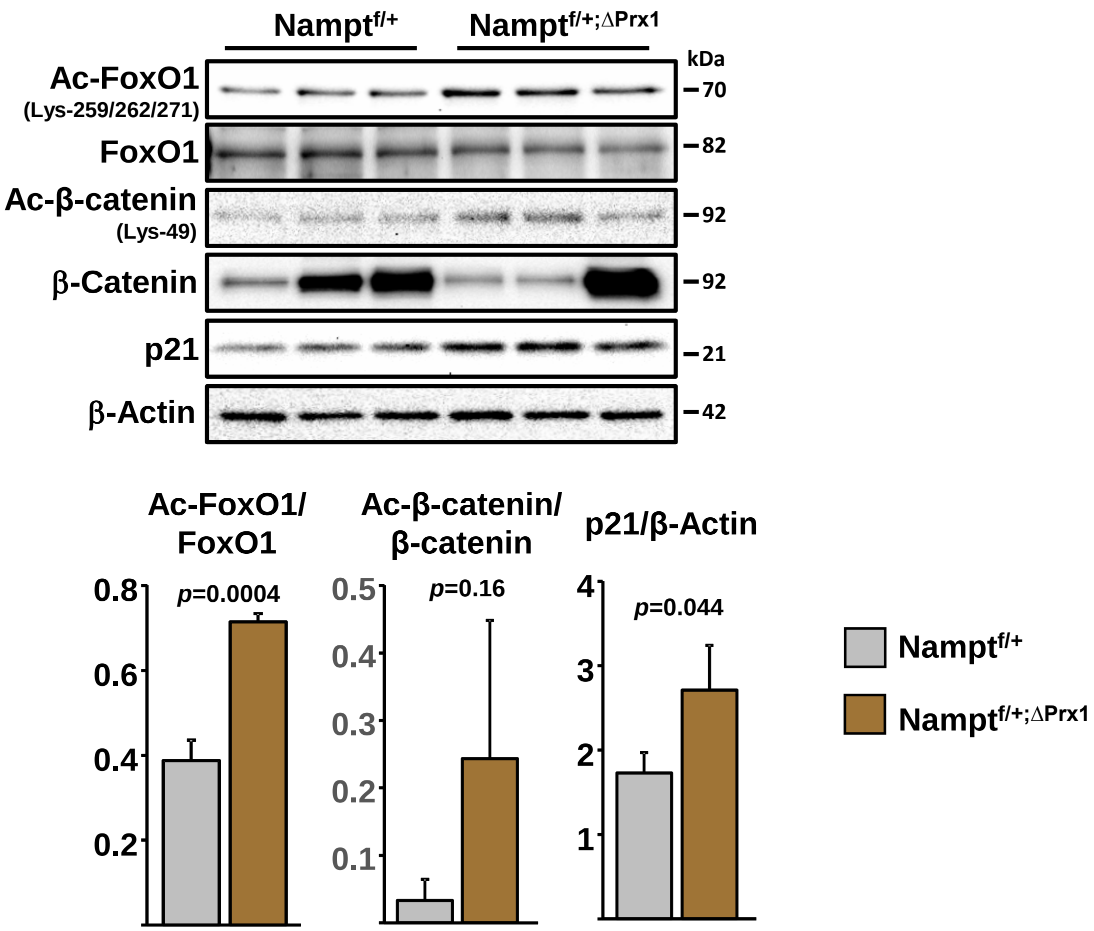

Nampt$^{f/+}$
Nampt$^{f/+;\Delta Prx1}$
kDa
Ac-FoxO1
(Lys-259/262/271)
70
FoxO1
82
Ac-β-catenin
(Lys-49)
92
β-Catenin
92
p21
21
β-Actin
42
Ac-FoxO1/ FoxO1
p=0.0004
0.8
0.6
0.4
0.2
Ac-β-catenin/ β-catenin
p=0.16
0.5
0.4
0.3
0.2
0.1
p21/β-Actin
p=0.044
4
3
2
1
Nampt$^{f/+}$
Nampt$^{f/+;\Delta Prx1}$

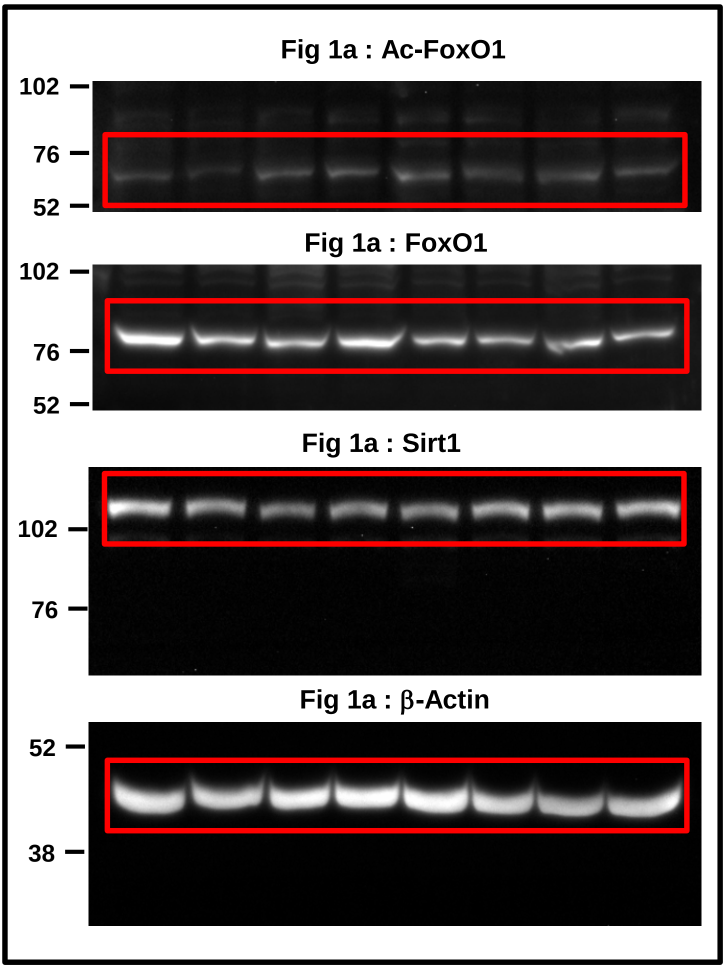
Fig 1a : Ac-FoxO1
102
76
52
Fig 1a : FoxO1
102
76
52
Fig 1a : Sirt1
102
76
Fig 1a : β-Actin
52
38

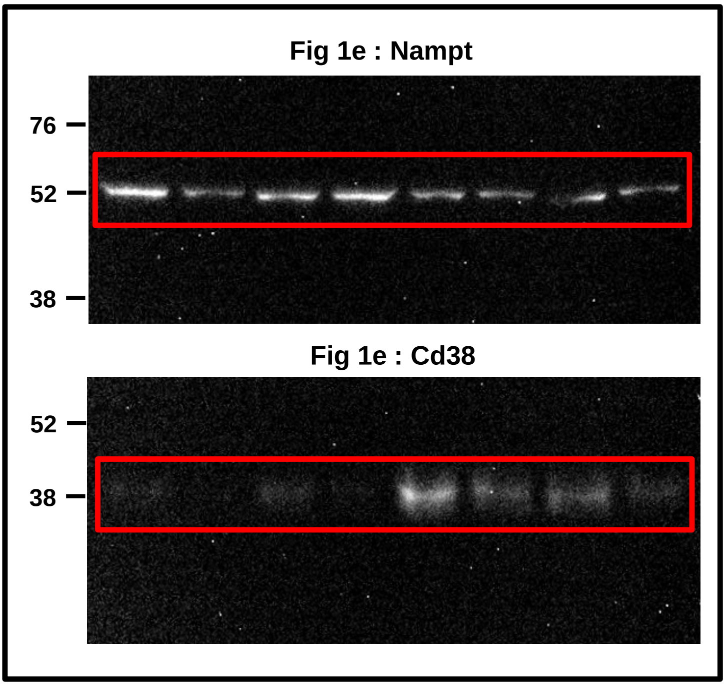
Fig 1e : Nampt
76
52
38
Fig 1e : Cd38
52
38

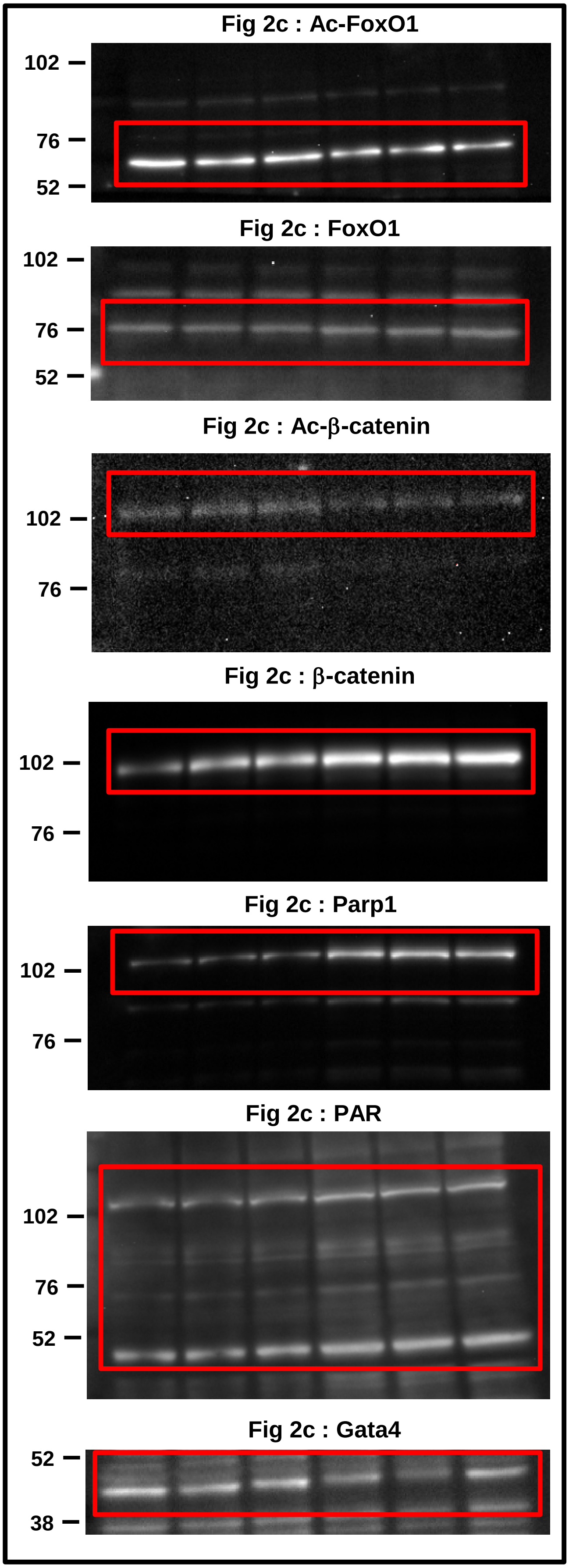
Fig 2c : Ac-FoxO1
102
76
52
Fig 2c : FoxO1
102
76
52
Fig 2c : Ac-β-catenin
102
76
Fig 2c : β-catenin
102
76
Fig 2c : Parp1
102
76
Fig 2c : PAR
102
76
52
Fig 2c : Gata4
52
38

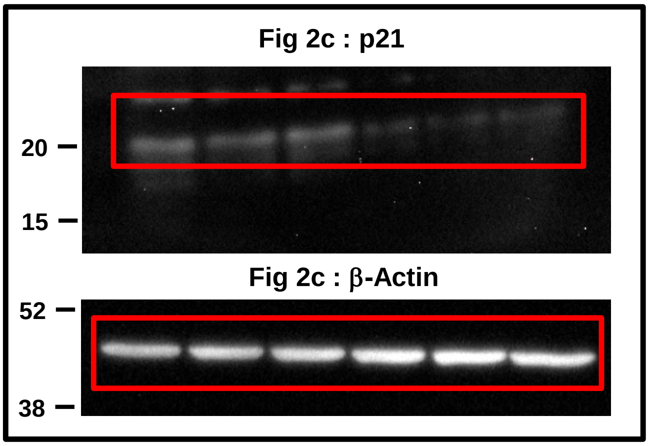
Fig 2c : p21
20
15
Fig 2c : β-Actin
52
38

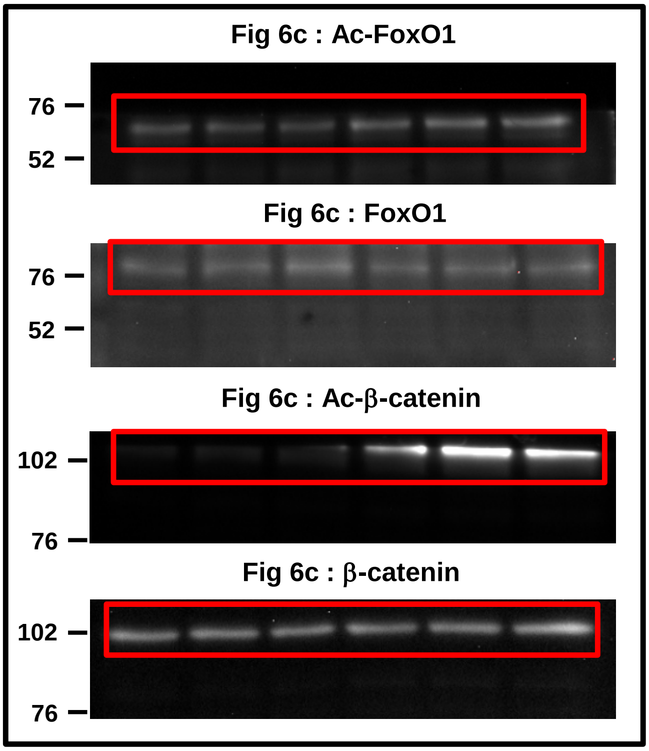
Fig 6c : Ac-FoxO1
76
52
Fig 6c : FoxO1
76
52
Fig 6c : Ac-β-catenin
102
76
Fig 6c : β-catenin
102
76

Supplementary Figure 1. Administration of NR to aging mice does not affect trabecular bone mass. Twelve-month-old C57BL/6 female mice were treated without or with NR, administered in the drinking water, until sacrifice at the age of 20 months. Trabecular bone volume (BV/TV) and microarchitecture in vertebrae L5 (n=15-18/group). Data represent mean ± S.D.; p values by two tail unpaired t-test.

Supplementary Figure 2. Mice haploinsufficient for Nampt in mesenchymal cells have normal trabecular bone. Male *Nampt*$^{f/+;\Delta Prx1}$ and *Nampt*$^{f/+}$ control littermates were generated and sacrificed at 2 (n=10 and 9, respectively) and 8.5 month of age (n=9 and 10, respectively). Trabecular bone volume (BV/TV) and microarchitecture in the distal end of the femur. Data represent mean ± S.D.; p values by two tail unpaired t-test.

Supplementary Figure 3. Deletion of Nampt promotes FoxO and β-catenin acetylation. Protein levels by Western blot in bone marrow-derived cells obtained from 8.5-mo-old male mice cultured for 5 days with osteogenic media (each lane represents one animal). Data represent mean ± S.D.; p values by two tail unpaired t-test.

Supplementary Figure 4. Lists of original gel images of western blot analysis.
Boxes highlight lanes used in figures.
